# Multiparametric magnetic resonance tomography and MRI/TRUS-fusion-biopsy for index lesion detection: correlation with radical prostatectomy specimen

**DOI:** 10.1186/1470-7330-15-S1-S1

**Published:** 2015-10-02

**Authors:** JP Radtke, C Schwab, MB Wolf, MT Freitag, C Alt, C Kesch, IV Popeneciu, C Huettenbrink, C Bergstraesser-Gasch, T Klein, S Duensing, S Roth, HP Schlemmer, M Roethke, M Hohenfellner, B Hadaschik

**Affiliations:** 1 Department of Urology University Hospital Heidelberg, Heidelberg, Germany; 2 Department of Radiology German Cancer Research Center Heidelberg, Heidelberg, Germany; 3 Department of Diagnostic and Interventional Radiology University, Hospital Dusseldorf, Dusseldorf, Germany; 4 Institute of Pathology University Hospital, Heidelberg, Germany

## Aim

Multiparametric MRI (mpMRI) and MRI-targeted fusion-biopsy (TB) detect significant prostate cancer (PC) more accurately than conventional biopsies alone. The aim of this study was to evaluate the detection accuracy by mpMRI and TB on radical prostatectomy (RP) specimen.

## Methods

We selected 120 consecutive patients who underwent transperineal fusion-biopsy before RP. All men received a saturation biopsy (SB) in addition to targeted biopsies of lesions with PIRADS≥2. On RP specimen, the index lesion was defined as highest Gleason score (GS) or highest tumour volume (TV). GS=3+3 and TV≥1.2ml or GS=3+4 and TV≥0.7ml or GS>3+4 were considered significant PC. We performed Spearmans correlation analysis between mpMRI and RP and Fisher`s test between mpMRI, TB and SB.

## Results

Overall, 120 index lesions and 71 non-index lesions were detected. 107 index and 51 non-index lesions harbored significant PC. MpMRI detected 110/120(91.7%) index lesions, while TB alone diagnosed only 96/120(80.0%) and SB alone 110/120(91.7%). The combination of SB and TB detected 115/120(95.8%) index foci. The combination of TB and SB outperformed TB alone (*p*=0.017) for detection of all significant PC. Additionally, TB performed significantly worse compared to SB alone for all significant tumour detection (*p*=0.034). Spearmans correlation coefficient for index lesion concordance between mpMRI and RP was 0.865(*p*<0.001). TB provided greatest benefit in men undergoing repeat biopsy.

## Conclusions

MpMRI detected 91.7% index lesions compared to RP. However, TB alone missed 21.5% of all significant foci. Thus, the combination of both biopsy approaches should be incorporated in the biopsy workflow to predict PC most accurately.

